# A sensitive fluorescence-based assay to monitor enzymatic activity of the essential integral membrane protein Apolipoprotein N-acyltransferase (Lnt)

**DOI:** 10.1038/s41598-019-52106-8

**Published:** 2019-11-04

**Authors:** Karine Nozeret, Alix Boucharlat, Fabrice Agou, Nienke Buddelmeijer

**Affiliations:** 1 Genetics and Biology of the Bacterial Cell Wall Unit, Institut Pasteur, 25-28 rue du docteur Roux, 75724 cedex 15, Paris, France; 2 INSERM group Avenir, Institut Pasteur, 25-28 rue du docteur Roux, 75724 cedex 15, Paris, France; 3 Chemogenomic and Biological Screening Platform, Center for Technological Resources and Research (C2RT), Institut Pasteur, 25-28 rue du docteur Roux, 75724 cedex 15, Paris, France

**Keywords:** Transferases, Chemical modification

## Abstract

Lipoprotein modification is an essential process in Gram-negative bacteria. The action of three integral membrane proteins that catalyze the transfer of fatty acids derived from membrane phospholipids or cleave the signal peptide of the lipoprotein substrate result in the formation of mature triacylated proteins. Inactivation of the enzymes leads to mis-localization of immature lipoproteins and consequently cell death. Biochemical studies and the development of *in vitro* assays are challenging due to the fact that the enzymes and substrates are all membrane-embedded proteins difficult to overproduce and purify. Here we describe a sensitive fluorescence-based assay to monitor bacterial apolipoprotein N-acyltransferase activity.

## Introduction

**Figure 1:**
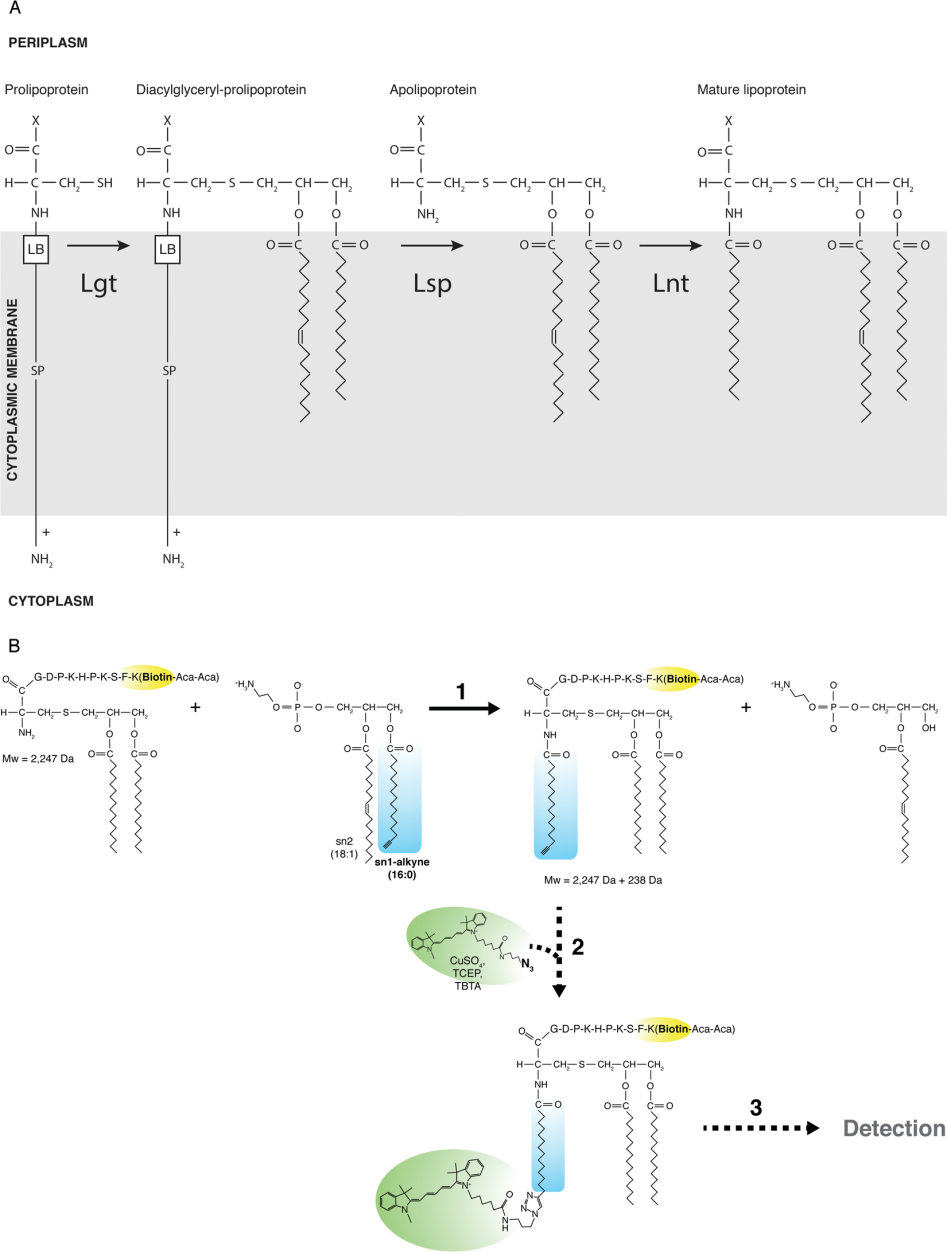
Schematic representation of lipoprotein modification in proteobacteria. (**A**) Sequential reactions in the cytoplasmic membrane catalyzed by Lgt, Lsp and Lnt result in the formation of triacylated proteins. The fatty-acid moieties are derived from membrane phospholipids. LB: lipobox or lipoprotein recognition sequence, SP: signal peptide. (**B**) Fluorescence-based apolipoprotein N-acyl transferase assay: 1) Lnt catalyzes the transfer of C16:0(alkyne) onto FSL-1-biotin, 2) a click chemistry reaction with an azido-Cy5 results in conjugation of the fluorescent group onto the alkyne fatty acid, and 3) detection of product formation by in-gel fluorescence, Western blot and fluorescence spectroscopy on streptavidin coated 96-well plates. The biotin moiety on FSL-1 is highlighted in yellow, the transferred alkyne-fatty acid is highlighted in blue and the fluorescent group (Cy5) in green.

The lipoprotein modification pathway is essential for viability in bacteria. In Gram-negative (proteo-) bacteria, three integral membrane proteins catalyze the formation of triacylated proteins; prolipoprotein diacylglyceryl transferase (Lgt), signal peptidase II (Lsp) and apolipoprotein N-acyltransferase (Lnt)^1^ (Fig. 1A). Lgt and Lnt use membrane phospholipids as acyl donor and prolipoprotein and apolipoprotein (S-diacylglyceryl protein) as protein substrate in the reaction, respectively. Lgt catalyzes the transfer of a diacylglyceryl group from phosphatidylglycerol resulting in the formation of diacylglyceryl-prolipoprotein and glycerol-1-phosphate. Lsp then cleaves the signal peptide resulting in the formation of apolipoprotein. We have shown that Lnt catalyzes a two-step reaction via a ping-pong mechanism, whereby in the first step, a stable thioester acyl-enzyme intermediate is formed upon hydrolysis of phospholipid^2^. 1-Palmitoyl-2-oleoyl-*sn*-glycero-3-phosphatidylethanolamine (POPE) is the preferred substrate for Lnt^3,4^. When the lysophospholipid by-product is released, N-acyl transfer onto apolipoprotein occurs in the second step of the reaction^2,5^. We have recently solved the X-ray crystal structure of Lnt of two bacterial species, *Escherichia coli* and *Pseudomonas aeruginosa*, using the lipid cubic phase technology for membrane proteins^6^. The data showed that Lnt is embedded in the cytoplasmic membrane via eight transmembrane segments on top of which sits the nitrilase-like catalytic domain with catalytic triad E267-K335-C387 located in the periplasm. Several flexible loops containing essential residues face away from the active site and are thought to play a role in substrate entry and exit^5,6^. The structure of an inactive variant of Lnt shows how the enzyme might accommodate and lift-up lipids from the phospholipid bilayer. Highly similar X-ray crystal structures of Lnt of *E*. *coli* were reported by two other research groups at the same time^7,8^. The active site of the three lipoprotein modifying enzymes is oriented towards the periplasmic space, which is located between the cytoplasmic membrane and the outer membrane, thus allowing relatively easy access to small inhibitory molecules.

We developed an *in vitro* Lnt activity test based on the reduced mobility on Tris-Tricine Urea SDS PAGE of a small diacylglyceryl peptide upon N-acylation^3^. We showed that Lnt uses phospholipids with a small polar headgroup, carrying a saturated fatty acid on *sn*-1 (C16:0) and non-saturated fatty acid on *sn*-2 (C18:1), as acyl donor. Two *in vitro* activity assays have been reported for Lgt. Mao *et al*. used lipoGFP, a fusion protein between the first 24 amino acids of Lpp (Braun’s lipoprotein) of *E*. *coli* and GFP, as substrate for Lgt in a gel shift assay^9^. A second assay includes coupled enzymatic reactions that has been proposed to screen for Lgt inhibitors *in vitro*^10^. This approach is based on the conversion of glycerol-1-phosphate, the by-product of the Lgt reaction, into glycerol and phosphate by the addition of alkaline phosphatase and subsequent formation of dihydroxyacetone and NADH from glycerol by glycerol-dehydrogenase. The enzyme diaphorase together with NADH converts resazurin into a fluorescent product resorufin, which can be recorded using a fluorescence plate reader.

Alkyne fatty acids combined with bio-orthogonal chemistry has been described as a useful tool to study lipoprotein maturation in bacteria^11^. Here we describe an assay, based on our Lnt *in vitro* activity test^3^, to monitor apolipoprotein N-acyltransferase activity through direct read-out of fluorescent triacylated peptide using click chemistry (Fig. 1B). Product formation was analyzed by in-gel fluorescence and fluorescence spectroscopy in 96-well plate format. This sensitive assay allows detailed characterization of the molecular mechanism of acyltransferases and the development of a high-throughput-screen (HTS) set-up for screening of specific inhibitors.

## Results

### Various *FSL-1* peptide substrates are *N-acylated* by Lnt *in vitro*

**Figure 2:**
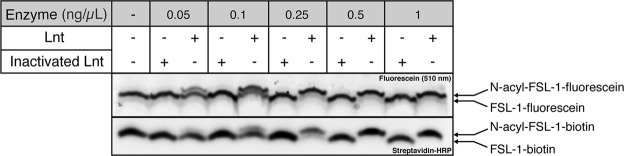
N-acylation of FSL-1 by Lnt. Lnt or heat-inactivated Lnt was added at different concentrations (0.05 ng/µL–1 ng/µL; 0.86 nM–17.2 nM) to a reaction mixture composed of POPE (PE-C16:0,C18:1 at 100 µM) and FSL-1-biotin or FSL-1-fluorescein (1 µM). Samples were incubated overnight at 37 °C and migrated on Tris-Tricine-Urea SDS-PAGE. FSL-1 biotin was detected on Western blot with Streptavidin-HRP and FSL-1-fluorescein was detected by in gel-fluorescence imaging at 510 nm. Images of the gel and Western blot were cropped using Image Lab software (Bio-Rad) to highlight the signal corresponding to FSL-1. Bands corresponding to substrate peptides and N-acylated products are indicated on the right. The experiment was performed in duplicate. Full-length gel and Western blot are presented in Supplementary Fig. S6.

In order to develop a sensitive method to assess Lnt activity *in vitro* we used different FSL-1 (fibronectin stimulating factor-1) substrates to monitor fluorescent read-out of the reaction. FSL-1 is a small decapeptide that contains a diacylglyceryl group at the N-terminal cysteine residue to mimic the natural apolipoprotein substrate that is extensively used as immune stimulating molecule through Toll-like receptor 2 (TLR2) signaling pathways^12^. FSL-1-fluorescein and FSL-1-biotin are both substrate in the Lnt reaction (Fig. 2). N-acyl transferase activity of Lnt results in a mobility shift due to a conversion of FSL-1 to N-acyl FSL-1 in the presence of 1-palmitoyl-2-oleoyl-*sn*-glycero-3-phosphoethanolamine (16:0-18:1 PE or POPE) phospholipid, which is the most efficient acyl donor^3,4^. Heat-inactivated Lnt was used as a negative control. This method was chosen to assure inactivation of both Lnt catalyzed steps; auto-acylation on active site residue C387 and N-acyl transfer onto apolipoprotein substrate (Fig. 1). The formation of N-acyl-FSL-1 is dependent on the concentration of Lnt, where complete conversion of substrate is observed at 0.5 ng/µL (8.6 nM) for both peptides upon overnight incubation at 37 °C.

FSL-1-fluorescein is more efficiently modified compared to FSL-1-biotin since the N-acyl FSL-1 product is clearly visible at low Lnt concentration, which makes it a promising substrate for analysis of N-acyl transfer by fluorescence polarization (FP). This method depends on the conversion of depolarized excited light into polarized emitted light due to changes in rotational correlation time of fluorescent molecules related to changes in molecular mass. Considering that the fluorescein label is rigid and the movement of the fluorophore closely reflects that of the monomer FSL-1 peptide, we hypothesized that the FP signal should be slightly enhanced when the FSL-1-fluorescein substrate (2,379 Da) is converted into N-acyl FSL-1 product due to the addition of the *sn*-1 fatty acid (palmitate; 238 Da) derived from the phospholipid. By in-gel fluorescence, Lnt activity was observed with wild-type enzyme, no activity was observed with heat-inactivated Lnt (Fig. S1). However, no difference in fluorescence polarization was observed in any condition, suggesting that a small increase in molecular mass was not sufficient as a measure for enzyme activity (Fig. S2). Biotinylated phosphatidylethanolamine (C12:0-biotin, C18:1-PE) was used as alternative acyl donor in the assay where transfer of the biotinylated *sn*-1 fatty acid of PE-biotin would lead to a significant increase in molecular mass of the product relative to palmitate. We first tested this phospholipid in the Lnt reaction using the gel-shift assay.

### PE-biotin is not a substrate for Lnt

**Figure 3:**
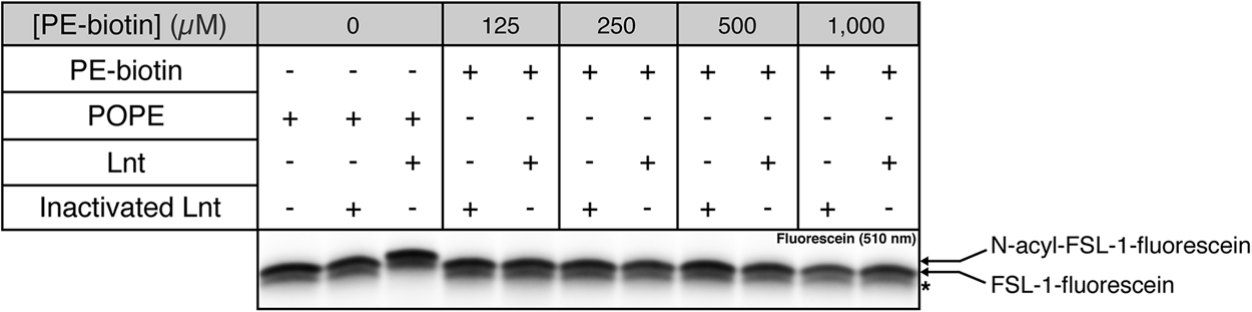
*In vitro* Lnt activity with PE-biotin as acyl donor. Lnt (0.5 ng/µL–8.6 nM) or heat-inactivated Lnt (0.5 ng/µL) was incubated in a reaction mixture composed of POPE (500 µM) or PE-biotin at different concentrations (125 µM–1,000 µM) and FSL-1-fluorescein (5 µM). Samples were incubated overnight at 37 °C and analyzed as described in Fig. 2. The experiment was performed in triplicate. A band indicated with an asterisk corresponds to a synthetic by-product of FSL-1. The image of the gel was cropped to highlight the signal corresponding to FSL-1-fluorescein. Full-length gel is presented in Supplementary Fig. S7.

**Figure 4:**
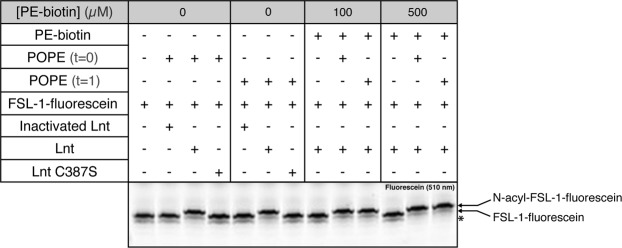
*In vitro* Lnt activity with PE-biotin in competition with POPE. Lnt (0.5 ng/µL–8.6 nM), heat-inactivated Lnt (0.5 ng/µL) or inactive enzyme Lnt (C387S) (0.5 ng/µL) was incubated with PE-biotin at different concentrations (100 µM–500 µM) with or without POPE (100 µM) and FSL-1-fluorescein (1 µM). At t = 0 both phospholipids were added simultaneously, at t = 1 POPE was added 1 hour after reaction in the presence of PE-biotin. Reactions were incubated overnight at 37 °C and analyzed as described in Fig. 2. The experiment was performed in triplicate. A band indicated with an asterisk corresponds to a synthetic by-product of FSL-1. The image of the gel was cropped to highlight the signal corresponding to FSL-1-fluorescein. Full-length gel is presented in Supplementary Fig. S8A.

PE-biotin was used in the Lnt reaction as acyl donor at various concentrations with FSL-1-fluorescein as substrate. Under these conditions, the product N-acyl-biotin-FSL-1-fluorescein is not detected, even at high concentrations of PE-biotin, contrary to the reaction with POPE in which mature lipopeptide is observed (Fig. 3). PE-biotin is either not a substrate or an inhibitor of Lnt. In order to discriminate between these two possibilities, we performed a competition assay with POPE and PE-biotin. POPE was added at the same time as PE-biotin (t = 0) or after 1 hour of reaction in the presence of PE-biotin (t = 1). In both conditions N-acyl-FSL-1-fluorescein is formed, suggesting that PE-biotin is not a competitive inhibitor for POPE (Fig. 4). This is independent of enzyme concentration since similar results are observed with higher Lnt concentration (1.0 ng/μL) (Fig. S3). Heat-inactivated Lnt and an inactive variant Lnt (C387S) are used as negative controls. Lnt (C387S) catalyzes the 1^st^ step of the N-acyl transfer reaction and forms a oxygen-ester acyl intermediate but is unable to transfer the acyl group onto apolipoprotein^2,8^. Both enzymes are unable to catalyze the N-acyl transferase reaction in the presence of POPE and no effect was observed with PE-biotin. PE-biotin is thus not a substrate for Lnt due to the presence of biotin on *sn*-1 that interferes with substrate recognition by the enzyme and cannot be explored as substrate in the fluorescence polarization approach. Altogether, this led us to propose the use of alkyne phospholipids produced by *E*. *coli* grown in the presence of alkyne fatty acids and subsequent click-chemistry to render both phospholipids and the N-acyl diacylglyceryl-peptide fluorescent.

### Use of Alkyne-PE as substrate and click-chemistry for fluorescent read-out of the Lnt reaction

**Figure 5:**
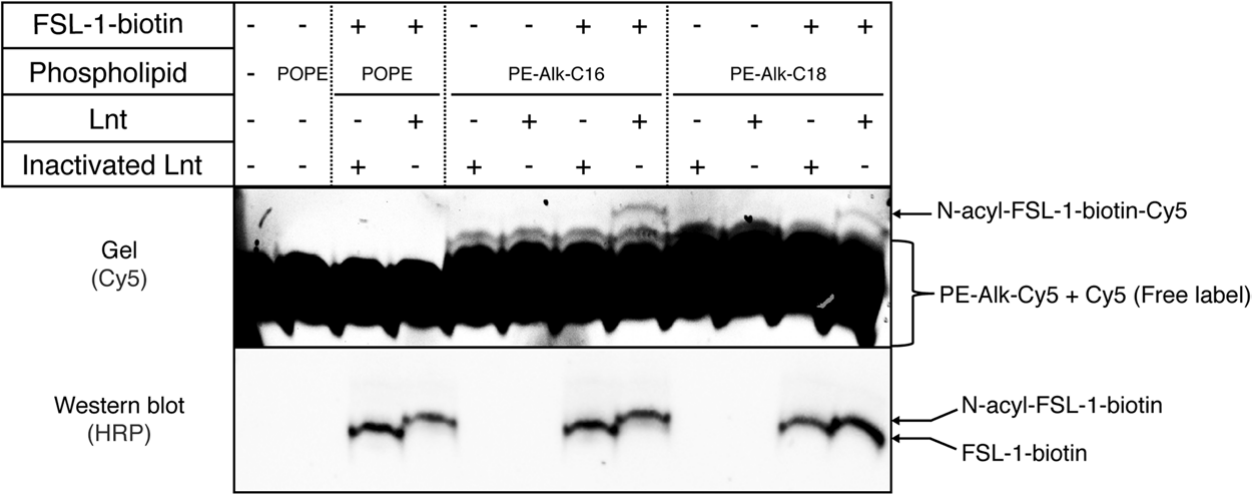
Lnt activity with PE-Alkyne from *E*. *coli* lipid extract. Formation of fluorescent N-acyl-FSL-1-biotin by a click chemistry reaction was performed after the transfer of *sn*-1 fatty acid from clickable PE catalyzed by Lnt. Two different PE-Alk phospholipids were used, PE-Alk-C16 and PE-Alk-C18. N-acyltransferase activity of Lnt was measured as a shift in mobility of FSL-1 peptide resulting from a conversion of FSL-1-biotin to N-acyl-FSL-1-biotin in the presence of a lipid donor. Lnt (1 ng/µL–17.2 nM) or heat-inactivated Lnt (1 ng/µL) was added to a reaction mixture composed of POPE (100 µM) or PE-Alk-C16 / PE-Alk-C18 (≅100 µM) containing endogenous non-alkyne PE and FSL-1-biotin (100 µM). The click-reaction was performed with 100 μM Azido-Cy5. The formation of fluorescent triacylated peptide was analyzed by in gel-fluorescence (top panel) and on Western blot in chemiluminescent mode (lower panel). The reagents, substrates and products are indicated on the right. Images of the gel and Western blot were cropped to highlight the signal corresponding to FSL-1-biotin. The contrast in the gel image was optimized using Image Lab (Bio-Rad) to improve detection of FSL-1-biotin. The experiment was performed in triplicate. Full-length gel and Western blot are presented in Supplementary Fig. S9.

Alkyne fatty acids have been successfully used to label and visualize phospholipids and lipoproteins by click-chemistry^11,13^. Bacterial cells were cultured in minimal medium in the presence of two types of alkyne fatty acids, palmitic acid alkyne (Alk-C16) or 17-octadecynoic acid (Alk-C18), to obtain alkyne phospholipids that were subsequently used as acyl donor in the Lnt reaction. The alkyne fatty acids are incorporated into phospholipids similar to endogenous fatty acids. Since the latter are also present, a mixture of phospholipid species with varying lipid composition is obtained and purified from membranes. We used thin-layer chromatography (TLC) to separate phospholipid species and to extract variants of phosphatidylethanolamine including alkyne modified PE^14^. The concentration of total PE was estimated on TLC relative to a known quantity of POPE, however, the concentration of alkyne-PE is difficult to measure in the mixture. PE-Alk-C16 and PE-Alk-C18 were tested as acyl donor in the Lnt reaction. After completion of the reaction, a Cu(I) catalyzed azide-alkyne cycloaddition reaction (CuAAC or click-chemistry) was used to label the alkyne-FSL-1-biotin product as well as alkyne-PE with an azide-containing fluorescent dye. The reactions were analyzed by in-gel fluorescence and Western blot. A fluorescent band corresponding to N-acyl-FSL-1-biotin-Cy5 is observed in reactions containing PE-Alk-C16 or PE-Alk-C18 and enzymatically active Lnt at the wavelength compatible with Cy5 (647 nm) (Fig. 5).

PE-Alk-C16 and PE-Alk-C18 differ in length of the fatty acid, which does not impact the N-acyl transfer and confirms our previous findings^3^. In the presence of POPE, N-acyl-FSL-1-biotin is formed but is not rendered fluorescent after click-chemistry since it does not contain an alkyne group. Similar results were obtained using azido-carboxyfluorescein (Azido-FAM) in the click-chemistry reaction (see below). Acylated product is also detected on Western blot using streptavidin coupled to horse-radish peroxidase (HRP) by chemiluminescence, demonstrating that the click reagents do not affect migration of substrate and product. These data show that Lnt activity can be visualized using fluorescence detection of triacylated peptide and validates the proof-of-principle of the assay.

### Optimization of the Lnt reaction with synthetic POPE-Alk

**Figure 6:**
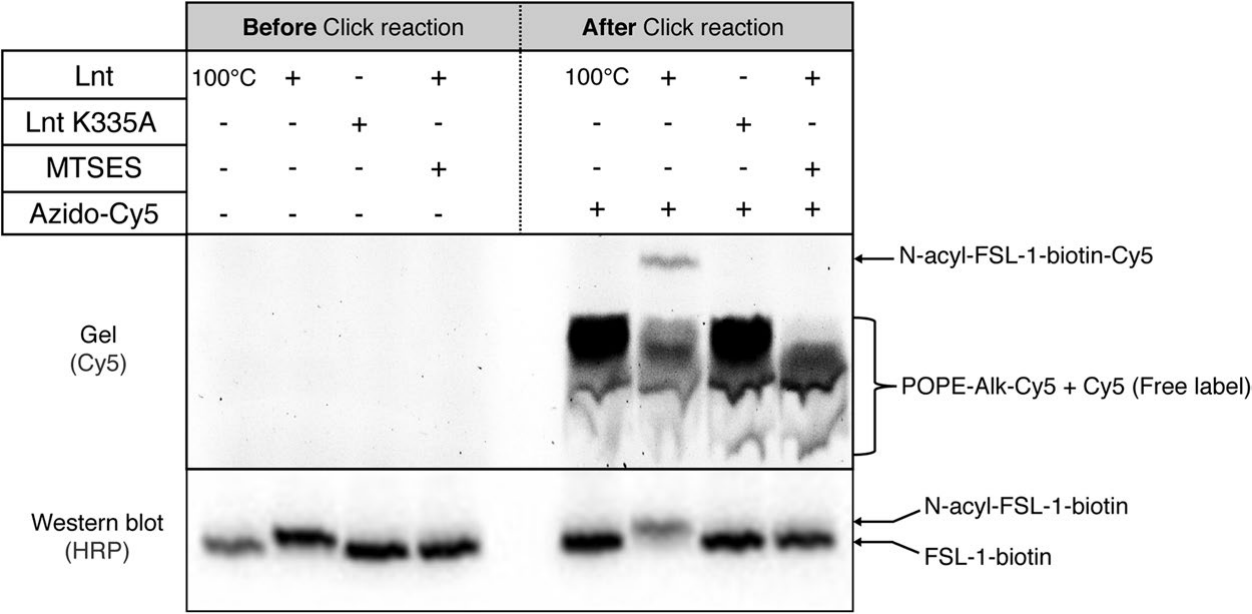
Lnt N-acylation activity with synthetic alkyne-POPE (POPE-Alk). Lnt (1 ng/µL–17.2 nM), heat-inactivated Lnt (1 ng/µL) or catalytic mutant Lnt (K335A) (1 ng/µL) was added to a reaction mixture composed of POPE-Alk (50 µM) and FSL-1-biotin (50 µM). Inhibitor MTSES was added at 10 mM to the Lnt reaction. An aliquot of samples was removed after an overnight incubation at 37 °C and analyzed by Tris-Tricine-Urea SDS-PAGE and immunoblotting with streptavidin-HRP (left panel). Then the click-reaction was performed with 50 μM Azido-Cy5 (right panel) and the formation of fluorescent triacylated peptide was verified on protein gel (top) and on Western blot (bottom) in chemiluminescence mode. Images were cropped to highlight the signal corresponding to FSL-1-biotin. The contrast in the gel image was optimized using Image Lab (Bio-Rad) to improve detection of the in-gel fluorescent signal. The experiment was performed in triplicate. Full-length gel and Western blot are presented in Supplementary Fig. S10.

To optimize the reaction conditions and to avoid use of a mixed population of PE molecules we next used synthetic *sn*-1-alkyne-POPE (POPE-Alk, Avanti Polar Lipids) as acyl donor in the Lnt reaction. We further optimized the concentration of enzyme, substrates and reagents to reduce the background signal due to free fluorescent label and non-used POPE-Alk substrate, and to increase the amount of triacylated product. The reaction was analyzed before click-chemistry to verify the use of synthetic POPE-Alk as acyl donor in the reaction by detection of product on Western blot. Currently, inhibitors of Lnt do not exist. We therefore used thiol specific reagent sodium (2-sulfonatoethyl)methanethiosulfonate (MTSES) as inhibitor of the reaction that blocks the thiol group of the active site cysteine in Lnt. A second inactive variant Lnt (K335A), that destabilizes the formation of the tetrahedral intermediate formed as part of the reaction^2^, was used as an additional negative control. N-acyl-FSL-1-biotin is observed when incubated with Lnt but not with heat-inactivated enzyme, nor with Lnt (K335A) or in the presence of MTSES (Fig. 6).

After click-chemistry a band is visible by in-gel fluorescence imaging (Cy5) corresponding to N-acyl-FSL1-biotin-Cy5, which is absent in the negative controls. Detection of N-acyl-FSL-1-biotin-Cy5 by chemiluminescence confirms the transfer of the *sn*-1 alkyne group.

### Fluorescence read-out on 96-well plates is compatible with HTS

**Figure 7:**
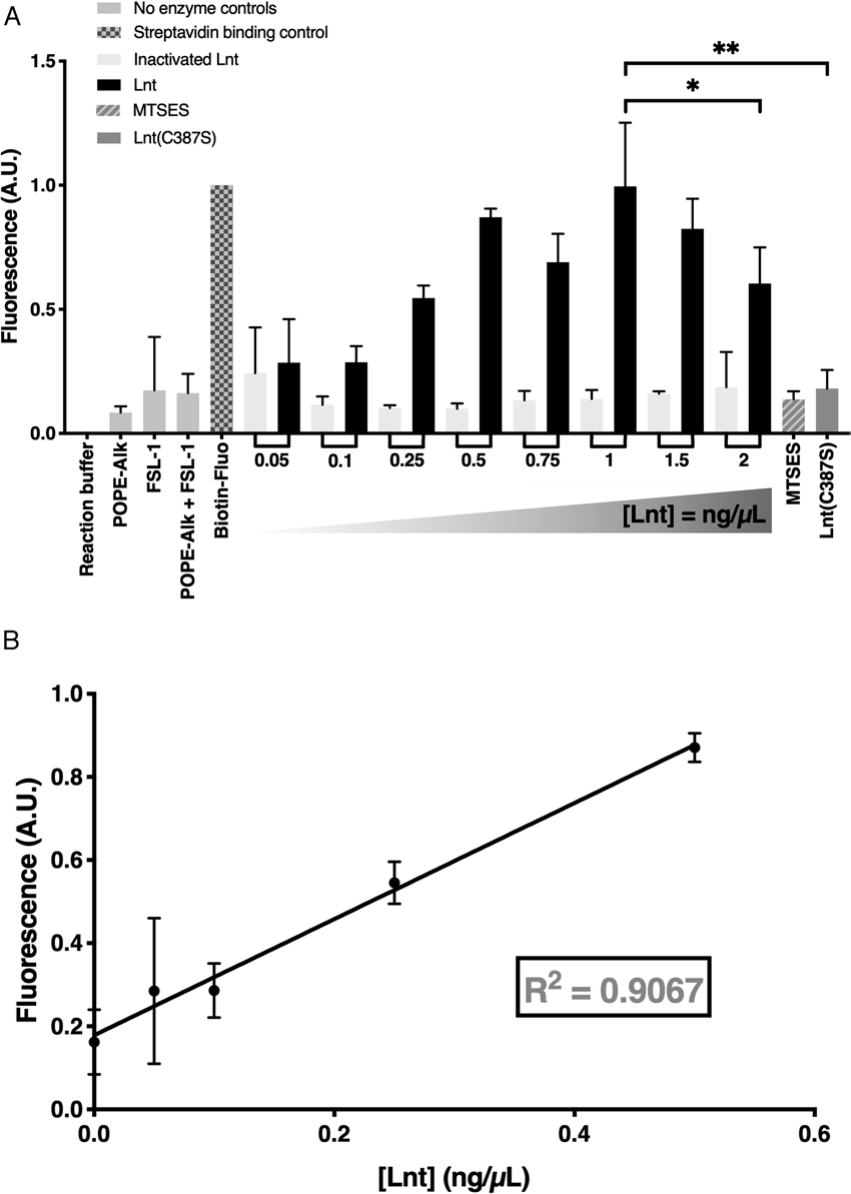
Detection of Lnt activity by fluorescence spectroscopy. (**A**) Fluorescence detection of N-acyl-FSL-1-biotin-fluorescein was performed on streptavidin coated 96-well plates. Reactions were incubated with various concentrations of enzyme up to 2 ng/μL overnight at 37 °C. Negative controls include heat inactivated Lnt (0–2.0 ng/μL), an active site mutant Lnt (C387S) (1.0 ng/μL) and inhibition of Lnt (1 ng/μL) in the presence of 10 mM MTSES. Reaction buffer contains Lnt buffer without substrate or enzyme nor click reagents, POPE-Alk only contains 50 μM POPE-Alk, POPE-Alk + FSL-1 contain both substrates at 50 μM each. Azido-FAM was used at 50 μM. Biotin-fluorescein (0.26 µM) was used as control for binding of biotin to home-made streptavidin coated plates and fluorescence detection at 524 nm in a Tecan M1000 Pro. Data shown is the average ± SD for n = 3 independent experiments. Fluorescence read-out is normalized to biotin-fluorescein sample. P-value calculated using Prism 8 for Lnt at 1.0 ng/μL and 2.0 ng/μL has a value of 0.033. The P-value for Lnt at 1.0 ng/μL and Lnt (C387S) is 0.0062. (**B**) Linear correlation between Lnt concentration and N-acyl transferase activity in the range of 0 ng/μL to 0.5 ng/μL. Data shown for n = 3 independent experiments.

The same experiment was performed using Azido-FAM in the click reaction, which is often used in standard fluorescence plate readers, and verified by gel electrophoresis. The samples were transferred onto home-made streptavidin-coated plates and analyzed by fluorescence. Biotin-fluorescein was used as control for binding and fluorescence detection. The concentration of biotin-fluorescein resulting in maximum non-saturating levels of fluorescence was empirically determined at 0.26 μM (Fig. S4). A 5-fold statistically significant increase in fluorescent signal is observed with Lnt compared to negative controls, including Lnt (C387S) and Lnt inhibited by MTSES (Fig. 7A). Maximum read-out is observed at 1 ng/μL enzyme and N-acyltransferase activity is inhibited by concentrations higher than 1.5 ng/μL (Fig. 7A). The assay is linear with Lnt concentrations up to 0.5 ng/μL (Fig. 7B). The optimum concentration of POPE-Alk is 50 μM (Fig. S5A). N-acyltransferase activity is linear in the range of 0–50 μM POPE-Alk and is inhibited at concentrations higher than 75 μM (Fig. S5A). FSL-1-biotin at 50 μM shows full activity with 1 ng/μL enzyme and 50 μM POPE-Alk and is constant up to 75 μM. A slight inhibition of N-acyltransferase is observed when 100 μM FSL-1-biotin is used (Fig. S5B). These results show that the optimal reaction conditions for the assay are 50 μM POPE-Alk, 50 μM FSL-1-biotin and 1 ng/μL Lnt incubated overnight at 37 °C for complete conversion of FSL-1-biotin. Background fluorescent is low due to the limiting amount of unused POPE-Alk substrate.

Easy and sensitive read-out of the apolipoprotein N-acyltransferase assay is a promising method to further characterize the molecular mechanism of the reaction and to compare enzymatic activities of various bacterial species.

## Discussion

Bio-orthogonal click-chemistry has been successfully used to visualize endogenous bacterial lipoproteins upon metabolic incorporation of alkyne fatty acids^11^. The short detection time by in-gel fluorescence and high sensitivity of this technique compared to the use of radioactive labeled fatty acids is an advantage concerning the time of exposure and waste issues. Furthermore, the fate of fatty acids in the eukaryotic cell has been followed using click-chemistry and the synthesis of various phospholipid species was determined and compared to classical radioactive labeling methods^13^. These studies suggest that alkyne fatty acids have the same functional property as building blocks of phospholipids as their natural counterparts.

Fluorescence polarization is a sensitive method to study protein-molecule interactions^15^. In non-viscous aqueous solutions, a small fluorescent molecule rapidly rotates resulting in depolarized emission. Upon binding to a macromolecule rotation will slow down, which results in polarized emission and consequently an increase in FP signal. The use of fluorescence polarization was not appropriate to accurately monitor Lnt activity. This is likely due to the formation of a complex mixture of mixed micelle-liposome structures that result from the presence of detergent, phospholipids as well as di- and triacylated peptide under our experimental conditions. Furthermore, even if we consider a rigid attachment of the fluorophore to FSL-1 we cannot rule out the possibility that the addition of palmitate to the peptide substrate is too small to induce a significant signal in fluorescence polarization.

In our fluorescence-based Lnt assay, the N-acyl peptide product was bound to streptavidin-coated plates after a click-chemistry reaction and fluorescence was measured as a direct read-out of the reaction (Fig. 1B). We found that the fluorescent signal is more than 5-fold enhanced in the presence of active enzyme compared to negative controls. Moreover, we showed that no triacyl product is formed when a bulky group such as biotin is attached to the extremity of the transferred fatty acid, indicating that our enzymatic assay is sensitive and can be used to select optimal acyl donor substrate for Lnt. The overall structure of Lnt from *E*. *coli* and *P*. *aeruginosa* is strikingly similar even though the primary sequence shows low identity^6^. This suggests that the mechanism of N-acyl transfer is likely to be conserved between bacterial species, which can now be easily addressed with the developed assay.

A HTS compatible *in vitro* assay using click chemistry was recently reported to identify inhibitors of Ras palmitoylation^16^. The principle of the reaction is based on the transfer of an acyl group (palmitate) from alkyne-acyl-CoA onto a small substrate peptide representing N-Ras catalyzed by membrane-bound S-palmitoyl transferase in vesicles (PAT). We used purified apolipoprotein N-acyltransferase (Lnt) under conditions that kept the protein in an active conformation^3^, which will likely reduce the chance of identifying inhibitors that interfere non-specifically with the assay such as detergent-like molecules as discussed in the screen for PAT inhibitors. A fluorescence-based screen using FRET was recently described to identify inhibitors of Lsp^17^. Direct fluorescent read-out in these methods is an advantage over the procedure proposed for Lgt where three enzymatic reactions are coupled that need to be controlled for, in order to avoid off-target hits^10^. In conclusion, the use of alkyne fatty acids and click-chemistry are powerful tools for the development of easy and sensitive assays to study acyl transfer reactions in detail. Furthermore, it could allow screening for specific inhibitors in an HTS screening campaign for the development of novel antibiotics.

## Methods

### Chemicals

Chemicals were obtained from Sigma-Aldrich, Fisher Chemical, Cell Signaling, Anatrace, Interchim, Calbiochem, Honeywell Research Chemicals or Acros Organics and were at least of analytical grade. Azido-FAM and Azido-Cy5 were obtained from Lumiprobe GmbH, Hannover, Germany. All phospholipids were from Avanti Polar Lipids, Alabaster, AL, USA. The FSL-1 lipopeptide derivatives were obtained from EMC Microcollections, Tübingen, Germany. Plasmid pASK-IBA3^+^, anhydrotetracycline, Streptactin^®^-Sepharose and desthiobiotin were purchased from IBA GmbH, Göttingen, Germany. All molecular biology enzymes were from New England Biolabs, Ipswich, MA.

### Overproduction of Lnt-Strep

Purification of Lnt was performed as described previously^3^ with slight modifications. PAP105 was used as an *E*. *coli* strain for protein production. Plasmid pCHAP9515 is a pASK-IBA3^+^ derivative containing the full-length *lnt* gene of *E*. *coli*. Plasmid pCHAP9519 contains a variant of *lnt* that resulted in the production of an inactive form of the enzyme Lnt (K335A)^3^, pCHAP9592 expresses *lnt* (C387S). This inactive variant of Lnt was constructed by site-directed mutagenesis using primers Cys387Ser_for (5′-GGTATTGAGCTTACTGCAGCTATT**AGC**TACGAGATCATTCTCGGCGAGC-3′) and Cys387Ser_rev (5′-GCTCGCCGAGAATGATCTCGTA**GCT**AATAGCTGCAGTAAGCTCAATACC-3′) with pCHAP9515 as template DNA. Base pair substitutions are shown in bold, a silent PstI restriction site (underlined) was introduced for screening of the desired mutant that was confirmed by sequencing. Strains were grown in 5 mL of Luria Broth (LB) with 100 µg/mL ampicillin from a single colony at 37 °C overnight. This preculture was used to inoculate 100 mL of LB with 100 µg/mL ampicillin to form a second preculture (overnight, 37 °C). This second preculture was used to inoculate 2 L of LB medium containing 0.1% glucose, and bacteria were initially grown at 30 °C under aeration at 180 rpm on a rotary shaker. At an OD_600_ = 0.6–0.8 (absorbance determined by a BioPhotometer Plus, Eppendorf), expression of *lnt* was induced by the addition of anhydrotetracycline (aTc) at 200 ng/mL. After 4–5 h and at an OD_600_ = 1.3–1.6, cells were harvested by centrifugation and washed with buffer Wa (20 mM Tris-HCl, pH 8, containing 150 mM NaCl and 0.5 mM EDTA). This buffer was used in all subsequent steps, and modifications are as indicated. The pellet was used directly for the preparation of membranes or stored at −20 °C.

### Membrane preparation and solubilization of Lnt

All steps were carried out at 4 °C. The frozen pellet was resuspended in 30 mL of buffer Wa with an EDTA-free Protease Inhibitor Cocktail (cOmplete™, Roche), 10 µg/mL DNase, 10 µg/mL RNase and 50 mM MgSO_4_. Cells were broken by three passages through a French pressure cell at 10,000 p.s.i. Unbroken cells and debris were removed by centrifugation at 13,000 × g for 20 min. The membrane fraction was prepared from the supernatant by ultracentrifugation at 120,000 × g for 60 min. The membrane pellets were either stored at −20 °C or immediately washed and resuspended in 12 mL of buffer Wa. n-Dodecyl β-D-maltoside (DDM) was added to a final concentration of 1% (v/v) to solubilize membrane proteins, and the suspension was incubated for 30 min with intermittent inversion on a rotation wheel. All insoluble material was removed by centrifugation at 120,000 × g for 60 min.

### Two-step purification of Lnt-Strep

The first step is by affinity chromatography on a Strep-Tactin Sepharose column using gravity flow. The column was equilibrated with 2 column volumes (CV) of buffer Wa and 2 CV of buffer Wb (Wa buffer containing 0.1% DDM). After binding 12 mL of the DDM-solubilized membrane fraction, the column was washed with 5 CV of buffer Wb. The protein was eluted from the column with 6 times 0.5 CV of buffer Wb containing 2.5 mM desthiobiotin.

In the second step, Lnt was purified by size exclusion chromatography using a prepacked Sephacryl High Resolution media HiPrep Sephacryl S400 HR column 16/60 (GE Healthcare) coupled to an Äkta Purifier FPLC system (GE Healthcare). The eluted fractions from the Strep-Tactin Sepharose column containing Lnt-Strep were loaded onto the gel filtration column that had been previously equilibrated in buffer Wc (Wa buffer containing 0.05% DDM) at a flow rate 0.5 mL/min.

Purity was evaluated after SDS-PAGE and staining with Coomassie Brilliant Blue, and the identity of the protein was confirmed by immunoblotting with anti-Strep antibodies. The protein concentration was determined by the bicinchoninic acid assay (BCA) using BSA as a standard (Pierce). Purified protein was stored in the elution buffer at −80 °C for at least 2 years at a concentration of 30–50 µg/mL without a detectable loss in activity.

### Lnt *in vitro* activity assay with POPE, PE-biotin, PE-alkyne or POPE-Alk

The *in vitro* Lnt activity assay has been described^3^ and is based on the reduced mobility of a small acylated peptide due to the addition of one fatty acid chain on a high-resolution Tris-Tricine-Urea SDS-PAGE system^18^. Here, N-acyltransferase activity of Lnt was monitored as a shift in migration of the lipopeptide FSL-1 conjugated either with biotin or fluorescein with a molecular weight (MW) of 2,247 Da or 2,379 Da, respectively. POPE (1-palmitoyl-2-oleoyl-*sn*-glycero-3-phosphatidylethanolamine), PE-biotin, membrane extracted PE-alkyne or pure synthetic POPE-Alk (1-hexadec-15-ynoyl-2-oleoyl-sn-glycero-3-phosphoethanolamine, Avanti Polar lipids) was used as acyl donor.

The standard assay for Lnt activity was carried out in a 20 µL reaction volume containing the reaction buffer composed of 50 mM Tris-HCl, pH 7.2, 150 mM NaCl, and 0.1% Triton X-100, phospholipids (50–1,000 µM) and FSL-1 (1–5 µM) and purified Lnt protein (0.86–17.2 nM). Heat-inactivated Lnt (100 °C, 5 min), inactive mutant Lnt (K335A) or Lnt (C387S) and the thiol-specific inhibitor MTSES were used as negative controls. Phospholipids were added as mixed micelles in 0.1% Triton X-100. Following a brief sonication and preincubation at 37 °C, Lnt was added. Samples were removed after overnight incubation at 37 °C and Lnt was rapidly inactivated by adding 5% SDS and heating to 100 °C. The relative levels of di- and triacylated peptide FSL-1 were monitored by SDS-PAGE and immunoblotting using streptavidin-HRP or fluorescein scans.

### Labelling of *E. coli* cells with alkyne fatty acids

*E*. *coli* K12 strain MG1655 was grown in minimal medium (M63 supplemented with 0.2% (w/v) glucose and 0.5% Vitamin B1 and 1 mM MgSO_4_). Bacterial cultures of OD_600_ = 1.7–2.0 were supplied with 20 μM Alk-C16 (palmitic acid alkyne) or Alk-C18 (17-octadecynoic acid) (from a 50 mM stock solution in DMSO). As a negative control, the same volume of DMSO was added. Cells were incubated at 37 °C for 3 h and pelleted at 4,000 × g for 5 min. Cells were washed twice with Phosphate Buffered Saline (PBS) and stored at −20 °C for lipid extraction.

### Total lipid extraction

The procedure for lipid extraction was performed according to Thiele *et al*.^13^. Briefly, the cell pellets were resuspended in a mixture of methanol (MeOH) and chloroform (CHCl_3_) (4:1), and the tube was briefly vortexed to obtain a single liquid phase with most of the cellular protein forming a precipitate. In case a two-phase mixture formed, MeOH was added dropwise until a single phase was observed. The tube was agitated for 1 h and centrifuged at 20,000 × g for 2 min, the supernatant was transferred to a new tube, and the pellet was discarded. A mixture of chloroform and 0.1% (v/v) aqueous acetic acid (1:2) was added followed by vortexing and centrifugation at 20,000 × g for 5 min. The upper aqueous phase was discarded, and the lower organic phase was transferred to a new tube and dried in a speed-vac. The dry lipid pellet was stored at −20 °C for future analysis.

### TLC separation and extraction of phospholipids

The thin layer chromatography plate (for analytical TLC: Merck Silica gel 60, non-fluorescent, 0.25 mm thick and for preparative TLC: Merck Silica gel 60, non-fluorescent, 2 mm thick, glass plate) was equilibrated in acetone before use. The lipid extract was dissolved in 50 µL of CHCl_3_ and applied onto the TLC plate before the development in a mixture of dichloromethane (DCM), MeOH and water (65:28:4) until the solvent front was 2–4 cm from the top. The plate was dried for 2 min under a hood to evaporate excess solvent. Next, the lipids were either stained in case of reference spots of PE to correlate migration or extracted from the TLC plate in case of purification of PE-alkyne. Lipids containing amine groups were stained with ninhydrin (Ninhydrin spray Sirchie, USA), spots of phosphatidylethanolamine (PE) appear as pink/purple. For the extraction of PE^14^, the areas of the silica gel containing the PE was scraped with a scalpel blade and the silica gel was transferred to a centrifuge tube. Each sample was eluted from the silica gel by resuspending the powder in eluting solvent through gently tapping the tube. The first and second elution were performed with the developing solvent DCM: MeOH: water (65:28:4) using a volume of 6 and 4 mL respectively, by subsequent vortexing and sonication. After centrifugation, the supernatant was collected. The third elution was made with 4 mL of MeOH, vortexing, sonication, centrifugation and collection of the supernatant. The fourth elution was obtained with 4 mL of MeOH: acetic acid: water (94:1:5). All supernatants were gathered, filtered on a Pasteur pipette with a cotton plug to eliminate silica particles, evaporated in a speedvac and stored at −20 °C.

### CuAAC or click chemistry reaction

A mixture of 50 or 100 μM Azido-Cy5/FAM (10 mM stock solution in DMSO); 1 mM tris(2-carboxyethyl)phosphine hydrochloride (TCEP) (50 mM freshly prepared stock solution in ddH_2_O); 0.2 mM tris[(1-benzyl-1H-1,2,3-triazol-4-yl)methyl]amine (TBTA) (2 mM stock in a mixture of tertbutanol and DMSO (4:1)); 1 mM CuSO_4_-5H_2_O (50 mM freshly prepared stock in ddH_2_O) was added to the 20 µL Lnt reaction solutions and samples were incubated at room temperature for 1 h. After incubation, an aliquot of each sample was diluted in loading buffer and heated at 100 °C for 5 min and loaded onto home-made Tris-Tricine SDS-PAGE gels for separation of peptides or processed for detection by fluorescence spectroscopy.

### Tris-Tricine SDS-PAGE and Immunoblotting

Peptides were separated in high-resolution Tris-Tricine-Urea SDS-PAGE gels consisting of three sequentially polymerized layers: (i) resolving gel 20% containing 6 M urea; (ii) spacer gel 11% with 4 M urea and (iii) stacking gel 4%^18^. Gels migrated at room temperature in a Mini-PROTEAN^®^ Tetra Vertical Electrophoresis Cell (Bio-Rad) at 30 V (initial), 50 V (next) and 75 V (final). FSL-1-biotin or FSL-1-fluorescein bands were transferred onto a nitrocellulose membrane by a Trans-Blot® Turbo™ Transfer System (Bio-Rad) and hybridized against streptavidin peroxidase (HRP) conjugate (for FSL-1-biotin) or directly analyzed by fluorescent detection (for FSL-1-fluorescein). SuperSignal™ West Femto Maximum Sensitivity Substrate Western blotting detection reagent (Thermo Scientific) was used as substrate. Chemiluminescent and fluorescent signals were detected with a Chemidoc MP gel imaging system (Bio-Rad).

### Monitoring product formation in 96-well plate format by fluorescence spectroscopy

Home-made streptavidin-coated microplate were obtained by incubation of 100 μL of 10 μg/mL streptavidin per well on 96-well plates (Greiner). After overnight incubation at 37 °C, plates were rinsed three times with 200 µL of PBST1 (PBS containing 0.05% Tween-20). Click chemistry samples containing the biotin-conjugated peptide FSL-1 (20 μL) were diluted in PBST1 (80 μL) and added to each well of a 96-well plate. The plate was incubated at room temperature while shaking for 1 h in the dark to bind biotin-conjugates onto the streptavidin-coated surface, after which the wells were rinsed six times with PBST2 (PBS with 1% Tween-20), three times with PBST1 (0.05% Tween20) and three times with PBS. Fluorescent signals were measured with a Microplate reader Infinite^®^ M1000 pro (TECAN).

## Supplementary information

Supplementary data
