# Supplementary material for: A sensitive fluorescence-based assay to monitor enzymatic activity of the essential integral membrane protein Apolipoprotein N-acyltransferase (Lnt)
Source: Sci Rep. 2019 Nov 4;9:15978. doi: 10.1038/s41598-019-52106-8 (PMC6828757; doi:10.1038/s41598-019-52106-8)
Supplement: Supplementary file 1 — Supplementary data [file 41598_2019_52106_MOESM1_ESM.pdf]

A sensitive fluorescence-based assay to monitor enzymatic activity of the essential integral membrane protein Apolipoprotein N-acyltransferase (Lnt)

Karine Nozeret, Alix Boucharlat, Fabrice Agou and Nienke Buddelmeijer

Supplementary method

Fluorescence polarization

Fluorescence polarization measurements were taken on a microplate reader InfiniteR F500 (TECAN) using a 485 +/- 20 nm excitation filter and a 535 +/- 25 nm emission filter.

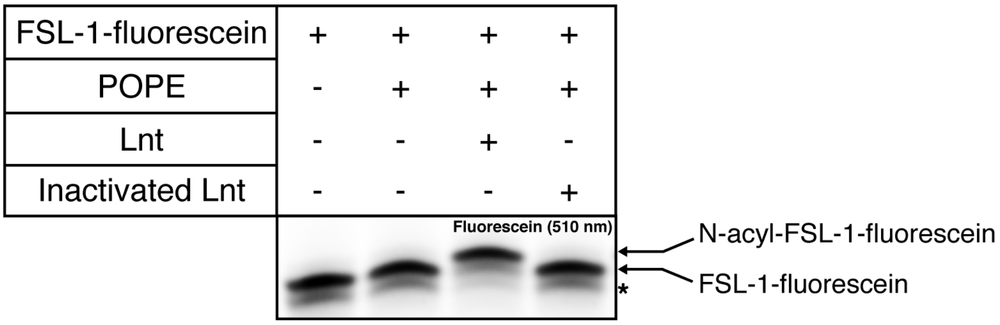

Figure S1

Figure S1. Detection of N-acyl FSL-1-fluorescein by in-gel fluorescence as control for fluorescence polarization.

Lnt (0.5 ng/µL – 8.62 nM) was added to a reaction mixture composed of POPE (PE-C16:0,C18:1 at 500 µM) and FSL-1-fluorescein (5 µM). Heat-inactivated Lnt (0.5 ng/µL) is used as negative control. Samples were incubated overnight at 37°C and migrated on Tris-Tricine-Urea SDS-PAGE. FSL-1-fluorescein was detected by in gel-fluorescence imaging at 510 nm. Substrate peptides and N-acylated products are indicated on the right. A band indicated with an asterisk corresponds to a synthetic by-product of FSL-1. The image of the gel was cropped using Image lab software (Bio-Rad). The experiment was performed in duplicate. Full-length gel is presented in Supplementary Figure S11.

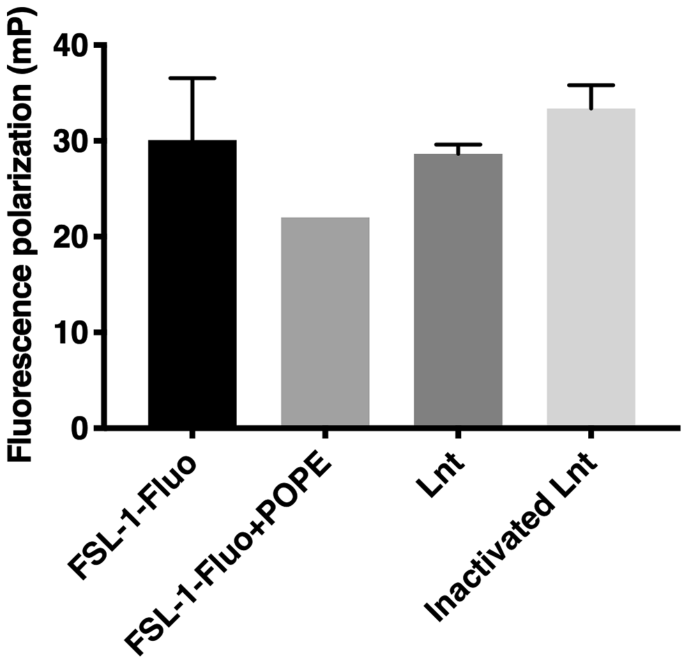

Figure S2

Figure S2. Detection of Lnt activity by fluorescence polarization

Fluorescence polarization signal of N-acyl-FSL-1- fluorescein on 96-well plates of samples described in Figure S1. FSL-1-Fluo and FSL-1-Fluo + POPE correspond to substrate and substrate with acyl donor, respectively, without incubation with enzyme. Data shown for n=2 experiments.

[PE-biotin] (μM)	0				0			100			500		
PE-biotin	-	-	-	-	-	-	-	+	+	+	+	+	+
POPE (t=0)	-	+	+	+	-	-	-	-	+	-	-	+	-
POPE (t=1)	-	-	-	-	+	+	+	-	-	+	-	-	+
FSL-1-fluorescein	+	+	+	+	+	+	+	+	+	+	+	+	+
Inactivated Lnt	-	+	-	-	+	-	-	-	-	-	-	-	-
Lnt	-	-	+	-	-	+	-	+	+	+	+	+	+
Lnt C387S	-	-	-	+	-	-	+	-	-	-	-	-	-

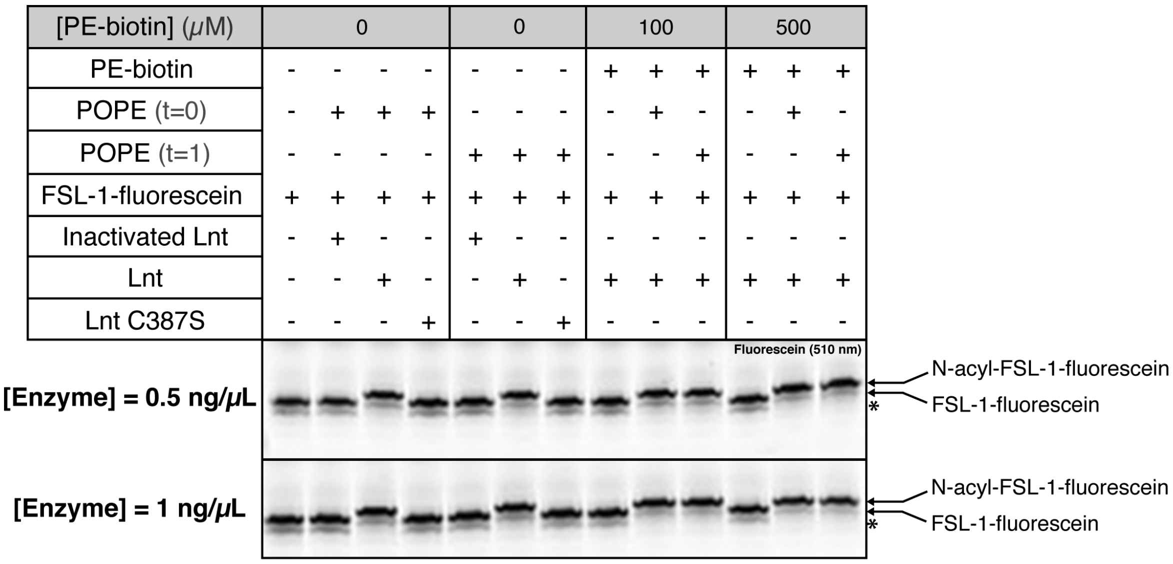

Figure S3

Figure S3. ***In vitro*** **Lnt activity with PE-biotin in competition with POPE.**

Comparison of 0.5 ng/μL and 1.0 ng/μL Lnt. The experiment was performed in triplicate.

Data with 0.5 ng/μL are shown in Figure 4.

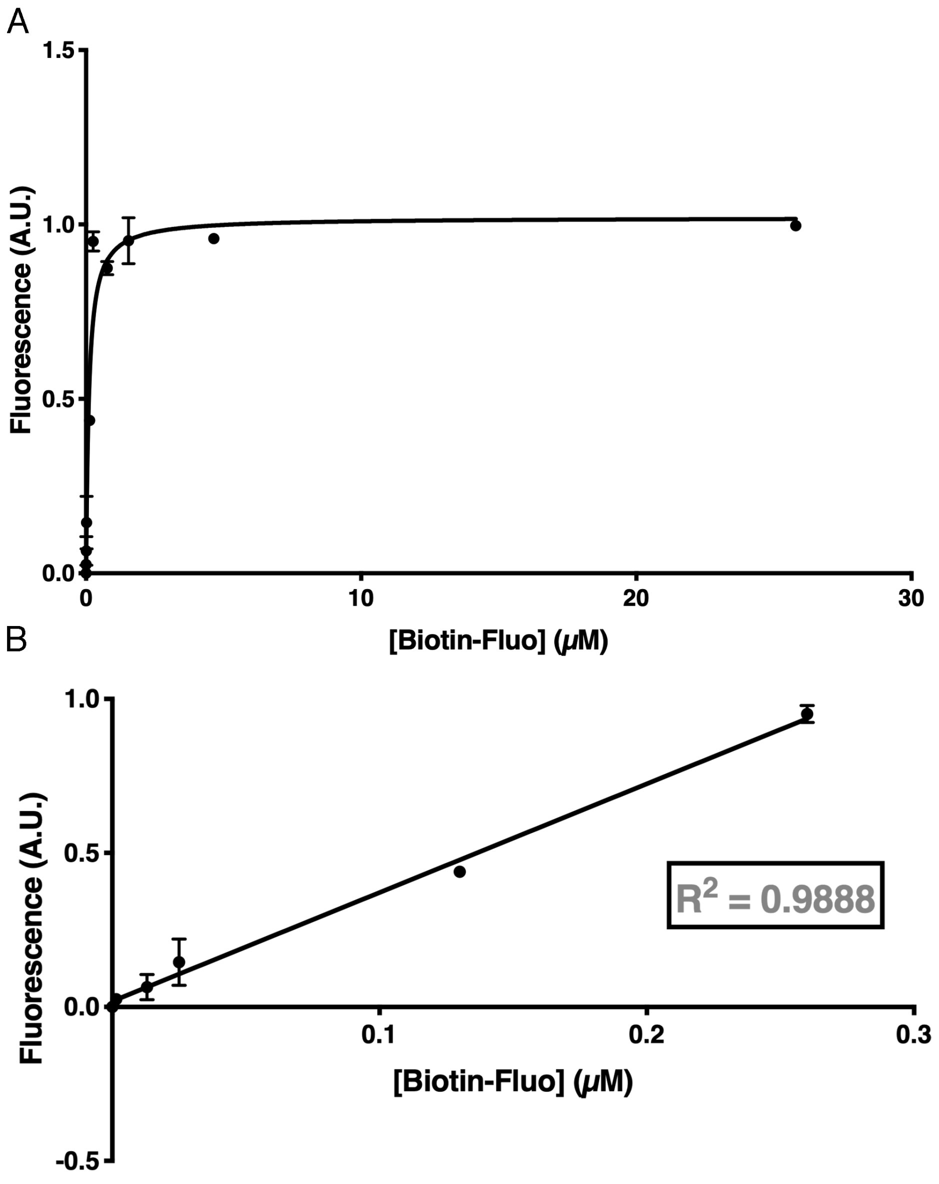

FIGURE S4

Figure S4. Detection of biotin-fluorescein by fluorescence spectrometry

Biotin-fluorescein was added to home-made streptavidin-coated 96-well plates in various concentrations and fluorescence was recorded at 524 nm. A. Fluorescence of biotin-fluorescein is plotted as function of biotin-fluorescein concentration (0 to 26 µM). B. A linear correlation between biotin-fluorescein concentration and fluorescence is observed from 0 to 0.26 µM. Data shown for n=2 experiments.

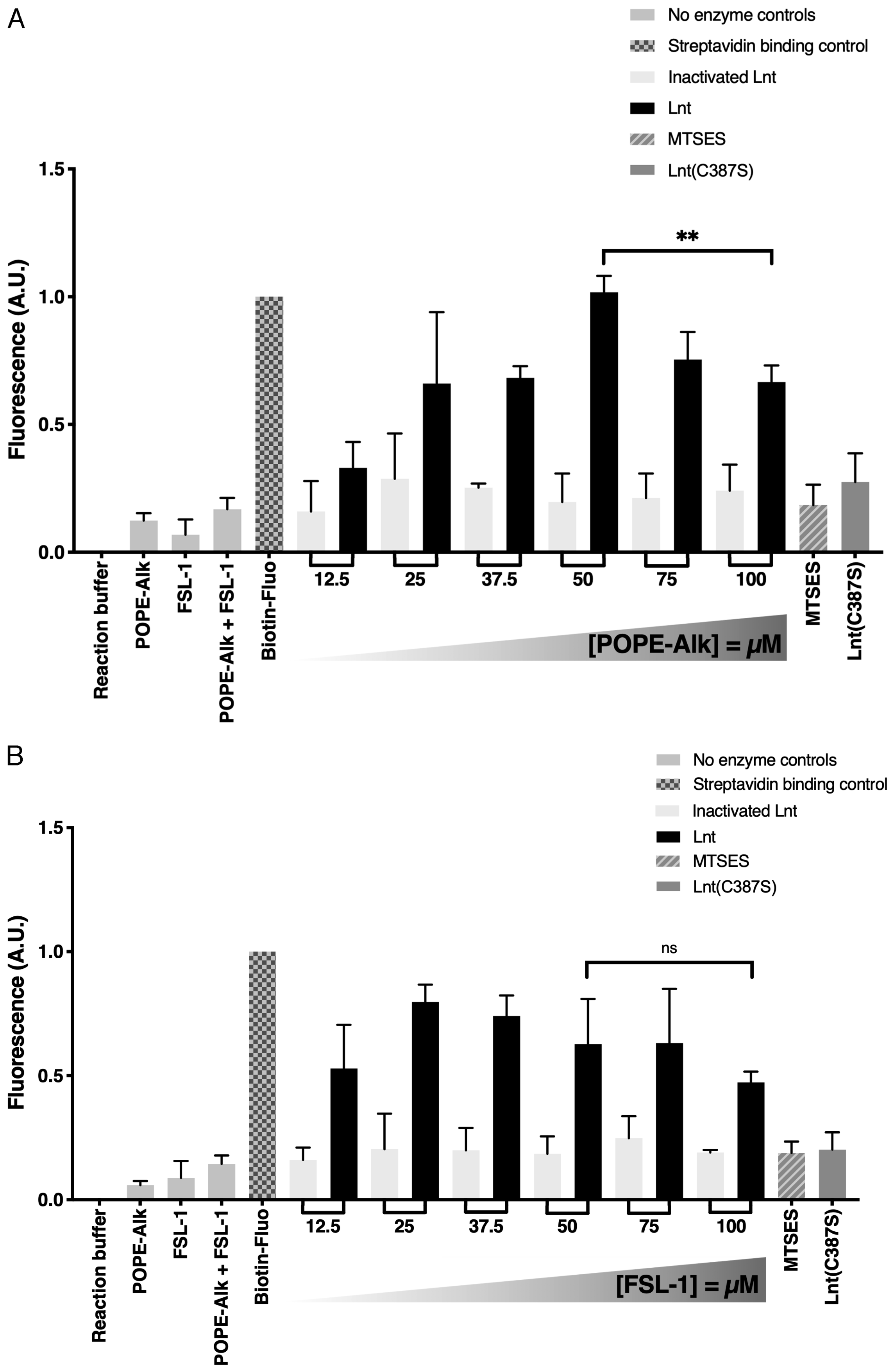
A
No enzyme controls
Streptavidin binding control
Inactivated Lnt
Lnt
MTSES
Lnt(C387S)
Fluorescence (A.U.)
1.5
1.0
0.5
0.0
**
Reaction buffer
POPE-Alk
FSL-1
POPE-Alk + FSL-1
Biotin-Fluo
12.5
25
37.5
50
75
100
[POPE-Alk] = μM
MTSES
Lnt(C387S)
B
No enzyme controls
Streptavidin binding control
Inactivated Lnt
Lnt
MTSES
Lnt(C387S)
Fluorescence (A.U.)
1.5
1.0
0.5
0.0
ns
Reaction buffer
POPE-Alk
FSL-1
POPE-Alk + FSL-1
Biotin-Fluo
12.5
25
37.5
50
75
100
[FSL-1] = μM
MTSES
Lnt(C387S)

Figure S5

Figure S5. Fluorescent read-out as function of substrate concentration

Reactions were incubated with Lnt at 1 ng/µL overnight at 37°C with varying substrate concentration. Click chemistry was performed with 50 µM azido-FAM and fluorescence measured at 524 nm by fluorescence spectrometry. A. POPE-Alk was added at different concentrations (0-100 µM) in the presence of 50 µM FSL-1-biotin. The *p*-value is 0.0027 for 50 µM and 100 µM POPE-Alk as calculated with Prism 8. B. FSL-1-biotin was added at different concentrations (0-100 µM) in the presence of 50 µM POPE-Alk. The *p*-value is 0.2242 for 50 µM and 100 µM FSL-1. Negative controls include heat-inactivated Lnt (1 ng/µL), an active site mutant Lnt (C387S) (1 ng/µL) and inhibition of Lnt (1 ng/µL) in the presence of 10 mM MTSES. Reaction buffer contains Lnt buffer without substrate or enzyme nor click reagents, POPE-Alk only contains 50 µM POPE-Alk, POPE-Alk+FSL-1 contain both substrates at 50 µM each. Biotin-fluorescein (0.26 µM) was used as control for binding of biotin to home-made streptavidin coated plates and fluorescence detection at 524 nm in a Tecan M1000 Pro. Data shown is the average ± SD for n=3 independent experiments. Fluorescence read-out is normalized to biotin-fluorescein sample.

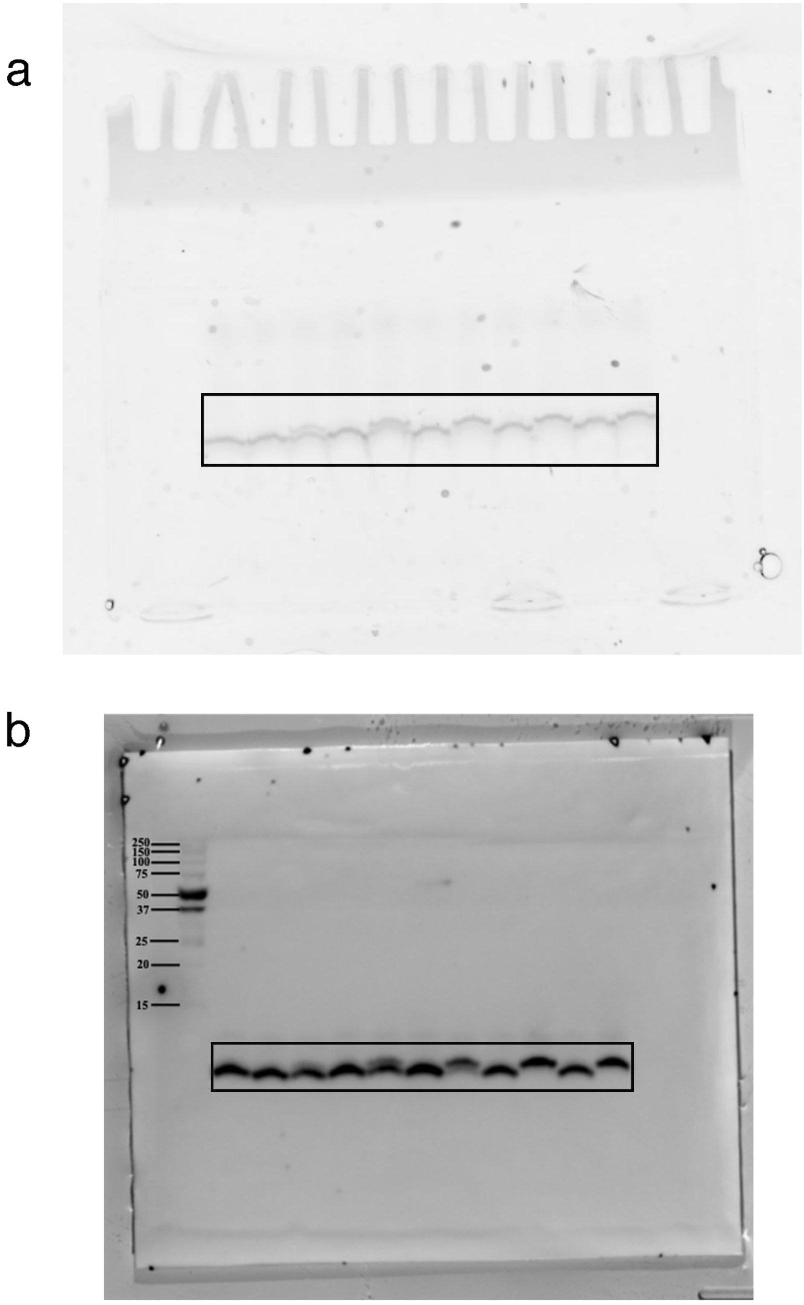

Figure S6

Figure S6. Full-length gel and Western blot shown in Figure 2.

A. In-gel fluorescence. B. Western blot with Streptavidin-HRP. Boxed areas are used for cropping.

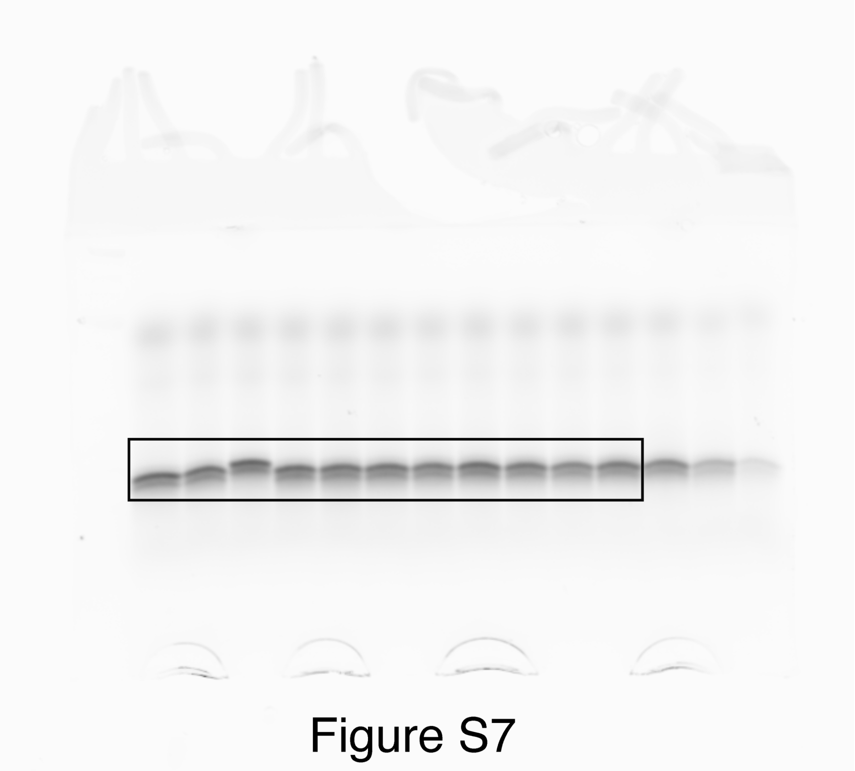

Figure S7. Full-length gel shown in Figure 3.

Boxed area is used for cropping.

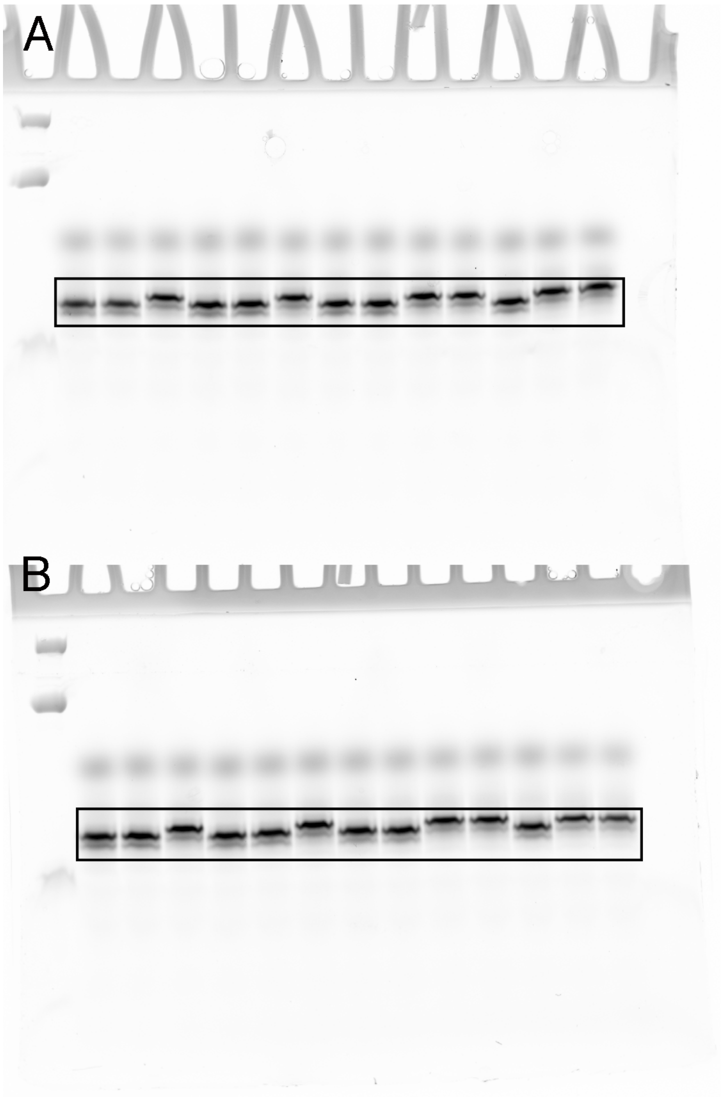

Figure S8

Figure S8. Full-length gels shown in Figure 4 and Figure S3.

A. Lnt at 0.5 ng/μL. B. Lnt at 1.0 ng/μL. Boxed areas are used for cropping.

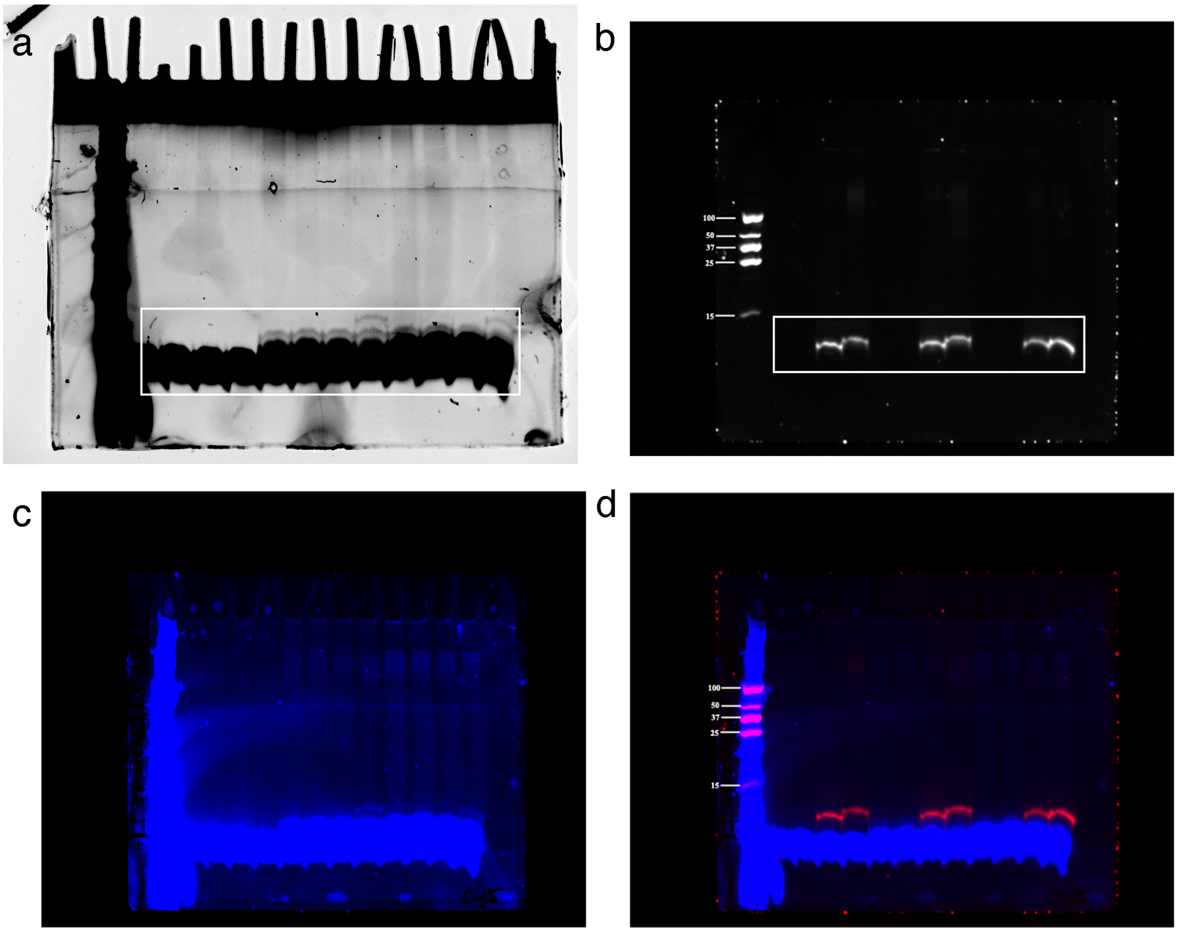

Figure S9

Figure S9. Full-length gel and Western blot shown in Figure 5.

A. In-gel fluorescence. B. Western blot with Streptavidin-HRP in chemiluminescence mode. C. Western blot in fluorescence mode (Cy5). D. Overlay image of panel B and C. Boxed areas are used for cropping.

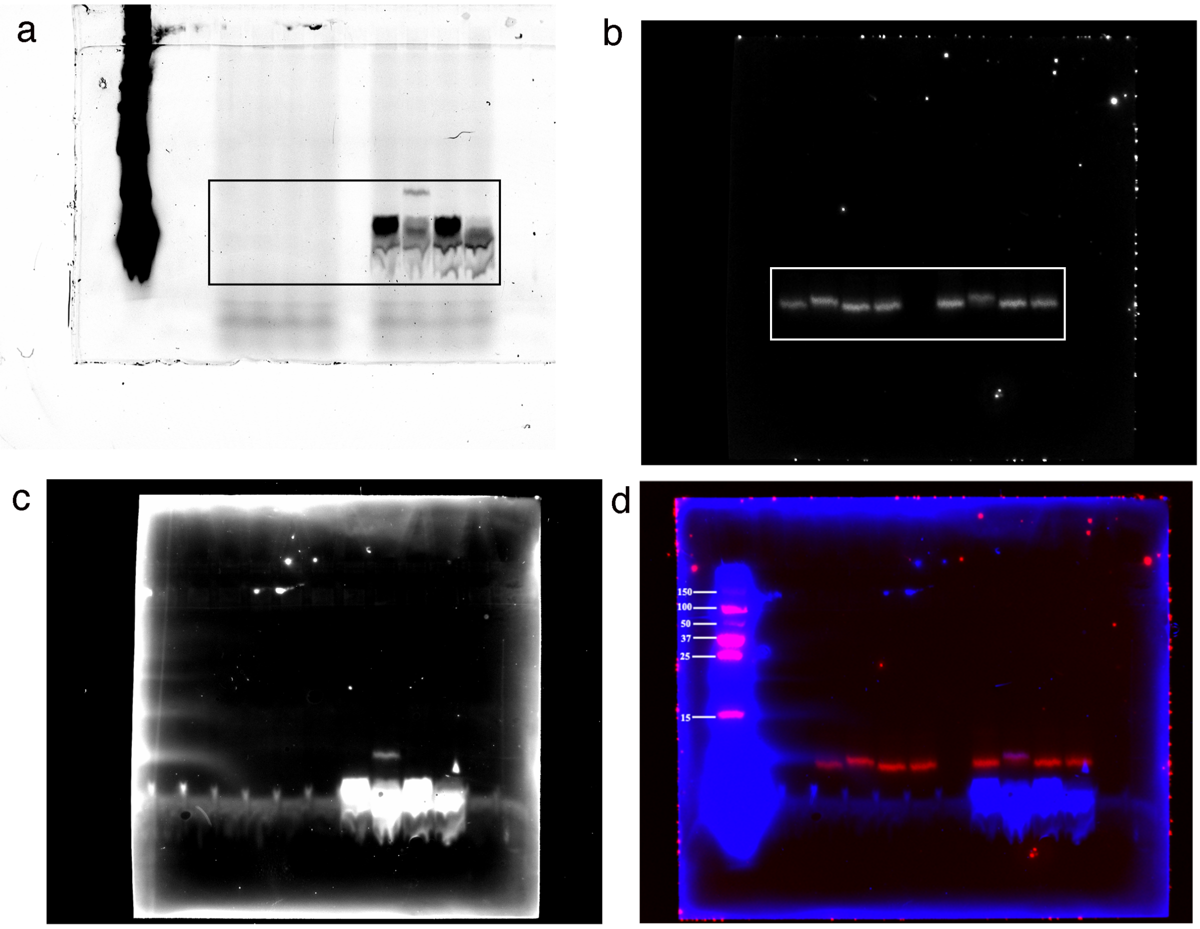

Figure S10

Figure S10. Full-length gel and Western blot shown in Figure 6.

A. In-gel fluorescence. B. Western blot with Streptavidin-HRP in chemiluminescence mode. C. Western blot in fluorescence mode (Cy5). D. Overlay image of panel B and C. Boxed areas are used for cropping.

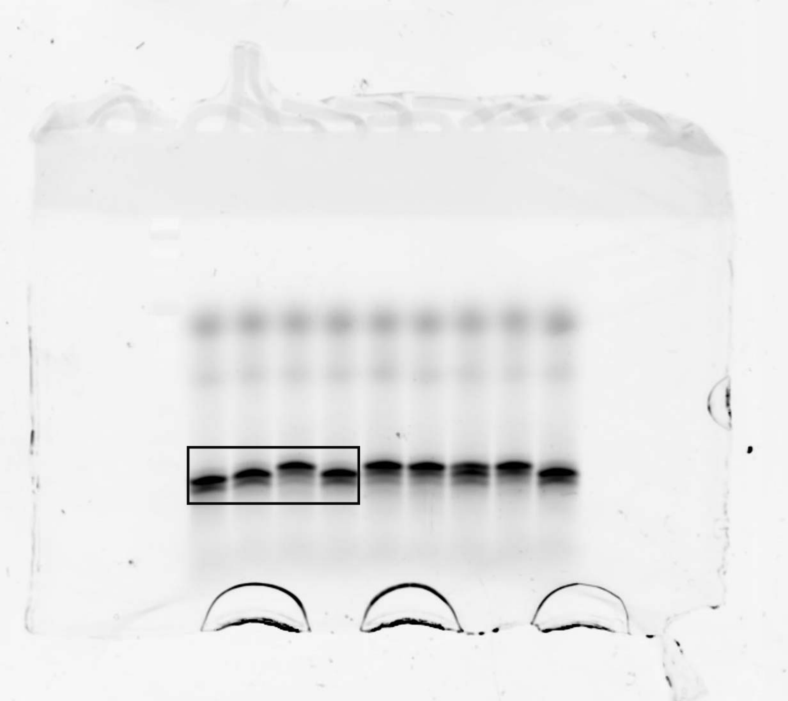

Figure S11

Figure S11. Full-length gel shown in Figure S1.

Boxed area is used for cropping.
